# Application of a Time-Stratified Case-Crossover Design to Explore the Effects of Air Pollution and Season on Childhood Asthma Hospitalization in Cities of Differing Urban Patterns: Big Data Analytics of Government Open Data

**DOI:** 10.3390/ijerph15040647

**Published:** 2018-03-31

**Authors:** Ching-Yen Kuo, Ren-Hao Pan, Chin-Kan Chan, Chiung-Yi Wu, Dinh-Van Phan, Chien-Lung Chan

**Affiliations:** 1 Institute of Information Management, Yuan-Ze University, 135 Yuan-Tung Road, Jung-Li, Taoyuan 320, Taiwan; jasmine@mail.tygh.gov.tw (C.-Y.K.); pan@51donate.com (R.-H.P.); johnny82128@gmail.com (C.-Y.W.); dvan2707@due.edu.vn (D.-V.P.); 2 Department of Medical Administration, Taoyuan General Hospital, Ministry of Health and Welfare, 1492 Zhongshan Road, Taoyuan Dist., Taoyuan 330, Taiwan; 3 Department of Pediatrics, Taoyuan General Hospital, Ministry of Health and Welfare, 1492 Zhongshan Road, Taoyuan Dist., Taoyuan 330, Taiwan; jean620104@yahoo.com.tw; 4 Innovation Center for Big Data and Digital Convergence, Yuan-Ze University, 135 Yuan-Tung Road, Jung-Li, Taoyuan 320, Taiwan; 5 University of Economics, The University of Danang, , 71 Ngu Hanh Son Street, Danang 550000, Vietnam

**Keywords:** childhood asthma hospitalization, air pollution, time-stratified case-crossover design, urban pattern, big data and open data

## Abstract

Few studies have assessed the lagged effects of levels of different urban city air pollutants and seasons on asthma hospitalization in children. This study used big data analysis to explore the effects of daily changes in air pollution and season on childhood asthma hospitalization from 2001 to 2010 in Taipei and Kaohsiung City, Taiwan. A time-stratified case-crossover study and conditional logistic regression analysis were employed to identify associations between the risk of hospitalization due to asthma in children and the levels of air pollutants (PM_2.5_, PM_10_, O_3_, SO_2_, and NO_2_) in the days preceding hospitalization. During the study period, 2900 children in Taipei and 1337 in Kaohsiung aged ≤15 years were hospitalized due to asthma for the first time. The results indicated that the levels of air pollutants were significantly associated with the risk of asthma hospitalization in children, and seasonal effects were observed. High levels of air pollution in Kaohsiung had greater effects than in Taipei after adjusting for seasonal variation. The most important factor was O_3_ in spring in Taipei. In children aged 0–6 years, asthma was associated with O_3_ in Taipei and SO_2_ in Kaohsiung, after controlling for the daily mean temperature and relative humidity.

## 1. Introduction

The World Health Organization estimated that 235 million people suffer from asthma worldwide [1]. Asthma is the most common chronic disease among children, and is also one of the major reasons for school absence, emergency medical treatment, and hospitalization during childhood. Research has indicated that asthma is responsible for 10 million missed school days per year in the USA [2]. In Taiwan, according to the National Health Insurance statistics, outpatient/emergency room visits or hospitalizations due to asthma totaled 1069 per 100,000 population in 1998, and increased to 3731 per 100,000 population in 2013, a three-fold increase in 15 years [3]. In the US, 10.5 million (14%) children have been diagnosed with asthma [4].

Many environmental factors have been linked to asthma causation [5], and it is necessary to identify environmental factors that could trigger an asthma attack. Children are known to be more sensitive to air pollution than adults [6,7], and a number of studies have already demonstrated that ambient air pollution contributes to childhood asthma morbidity [5,8,9]. In addition, children residing in urban communities experience particularly high incidence rates of asthma, and ambient air pollution levels have been found to be associated with hospitalization due to asthma [6]. However, different region-specific environmental factors may play important roles in the disease. Most previous studies were performed in a single city with a small sample size, and few studies have assessed the lagged effects of levels of different urban air pollutants and seasons on the incidence of asthma attack and asthma hospitalization in children over a long period of time in a large sample. 

We hypothesized that the different urban air pollutants and seasons have different effects of on asthma hospitalization. Therefore, this study aimed to investigate the association between hospitalization for childhood asthma and air pollution over a 10-year period using a large-scale database. We integrated the National Health Insurance Research Database (NHIRD) and air pollution and weather data from governmental open data using big data analysis methods. The objective of this study was to assess the impacts of environmental air pollution and season on hospitalization due to asthma for the first time in children between 2001 and 2010 in two different urban cities in Taiwan, Taipei, a business- and traffic-intensive city, and Kaohsiung, a large, heavily-industrial city, using a time-stratified case-crossover study design.

Taipei is the capital city of Taiwan, which sits at the northern tip of Taiwan; it has a population of approximately 2,702,000, and an average monthly temperature of 23.5 °C. Kaohsiung City is located in southern Taiwan, and is the second largest city on the island; it is characterized by heavy industry, with a population of approximately 2,770,000. Kaohsiung has a tropical monsoon climate, being dry in the winter, and hot and wet in the summer and autumn, with an average monthly temperature of 25.1 °C.

## 2. Materials and Methods

### 2.1. Asthma Hospitalization Data

**Figure 1:**
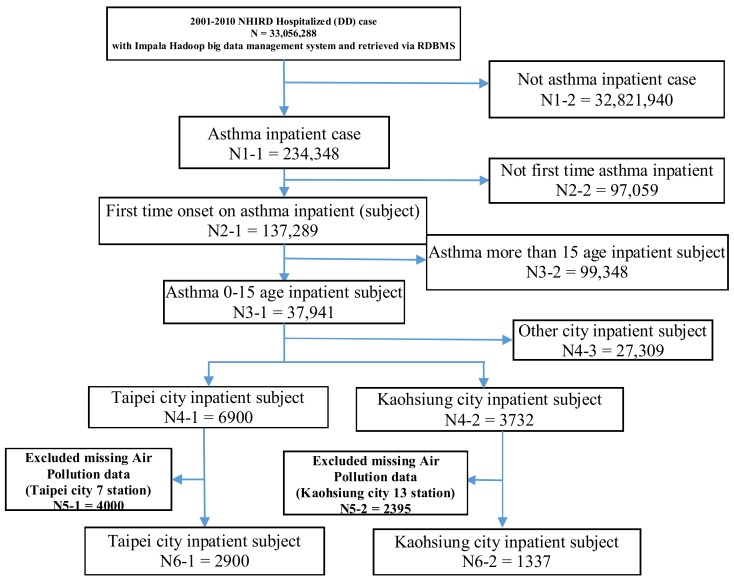
The study protocol.

This study was a retrospective population-based cohort analysis and ecological study of the associations between asthma hospitalization and different urban air pollutants and seasons. Childhood asthma hospitalization data were obtained from the National Health Insurance Research Database (NHIRD) established by the National Health Insurance Administration, Ministry of Health and Welfare, Taiwan. Taiwan launched a single-payer National Health Insurance program on 1 March 1995. As of 2014, 99.9% of Taiwan’s population were enrolled. The database of this program contains registration files and original claims data for reimbursement, and is maintained by the National Health Research Institutes (NHRI), Taiwan [10]. The NHIRD includes various data subsets, such as inpatient expenditure by admission (DD), details of inpatient orders (DO), ambulatory care expenditure by visit (CD), and details of ambulatory care orders (OO). In this study, we used the inpatient expenditure by admission DD data subset from 2001 to 2010, cases being identified when the ICD-9-CM code for asthma (493.XX) was listed as the major diagnosis in children under the age of 15. However, patients’ addresses were not available from the database, and therefore we assumed that a patient’s area of residence was close to the location of the hospital to which they were admitted. In order to avoid the confounding factor of readmission, from the registries of contracted medical facilities (HOSB) located in Taipei and Kaohsiung, first-time hospitalization events for asthma occurring from 2001 to 2010 were identified. The study protocol is shown in Figure 1.

### 2.2. Data Protection and Permission

Data in the NHIRD that could be used to identify patients or care providers, including medical institutions and physicians, is scrambled before being sent to the NHRI for database inclusion, and is further scrambled and encrypted before being released to each researcher. It is impossible to query the data alone to identify individuals at any level using this database. All researchers who wish to use the NHIRD and its data subsets are required to sign a written agreement declaring that they have no intention of attempting to obtain information that could potentially violate the privacy of patients or care providers. 

The study was of a retrospective cohort study design. The protocol was evaluated by the NHRI (Application and Agreement Number: NHIRD-104-183), who gave their agreement to the planned analysis of the NHIRD. Data protection and permission were also approved by the Institutional Review Board (IRB) of Taipei General Hospital, which has been certificated by the Ministry of Health and Welfare, Taiwan (IRB Approval Number: TH-IRB-0015-0003).

### 2.3. Air Pollution and Weather Data

Data on levels of air pollutants were obtained from Taiwanese Environmental Protection Administration air quality monitoring stations for the two cities: Taipei has 7 monitoring stations, and Kaohsiung has 13. Taipei City covers a total area of 271.7997 km^2^, and is divided into 12 administrative districts, the average size of which is 22.64 km^2^_._ Kaohsiung city covers a total area of 2951.85 km^2^, and is divided into 38 administrative districts, the average size of which is 77.68 km^2^_._ We selected air pollutant monitoring stations located in the same administrative division as the hospital to which patients were admitted. Each station takes hourly measurements of air pollutants, giving 24-h average daily concentrations of the following pollutants: particulate matter ≤2.5 μm (PM_2.5_), particulate matter ≤10 μm (PM_10_), ozone (O_3_), sulfur dioxide (SO_2_), and nitrogen dioxide (NO_2_). We excluded subjects admitted to hospitals that had no air quality monitoring station in the same administrative division or for which pollutant data were incomplete due to equipment failure or being under repair. 

The ambient daily temperature and relative humidity were used to control for meteorological conditions. Daily mean temperature and relative humidity data were provided by the Central Weather Bureau. Taipei has 14 monitoring stations, and Kaohsiung has 7. Because the temperature change within the same season is not so obvious in the same city, our weather variables data came from nearby hospital weather monitoring stations.

### 2.4. Statistical Analysis

We used a time-stratified case-crossover study design, which was proposed by Maclure [11] for the study of transient effects on the risk of acute events; it is characterized by the fact that each subject serves as his or her own control according to fixed individual characteristics, such as age, gender, lifestyle, socio-economic status, genetics and physiological status, etc. In this study, a case period was defined as the day of an asthma hospitalization, and the control period was when the patient did not experience a case-defining event; the control period was selected from other days of the same month and on the same day of the week as the case period. We used a two-week bi-directional approach with four control days in total (both one and two weeks before and after) that were matched to the case day.

Data were managed using the Impala Hadoop big data management system and retrieved via the RDBMS (Relational Database Management System), and conditional logistic regression analysis was performed using the software R, Version 3.3.2. Results are reported as odds ratios (ORs) and 95% confidence intervals (CIs) associated with an interquartile range (IQR) increase in PM_2.5_, PM_10,_ O_3_, SO_2_, and NO_2_ during the case day (lag day 0) and on each of the three days preceding asthma hospitalization (lag day l, lag day 2, and lag day 3). 

We chose the lag days based on prior literature [12], as this is the most common period that has been found to be significant in previous studies. All tests were conducted at a significance level of 0.05.

We performed stratified analysis by age group and season to control for seasonal effect. In the age-stratified analysis, the patients were stratified into three age groups: 0–6 years (preschool), 7–12 years (primary school), and 13–15 years (junior high school). Modified effects of season were examined using a four-level indicator variable for spring (March until May), summer (June until August), autumn (September until November), and winter months (December until February).

A single pollutant model and two-pollutant model were designed and adjusted for potential confounding factors, such as daily mean temperature and relative humidity.

## 3. Results

### 3.1. Hospitalization Characteristics

Table 1 presents the characteristics of the children admitted to hospital due to asthma during the study period. In total, there were 2900 first-time hospitalizations of children aged 0–15 years due to asthma in Taipei, and 1337 in Kaohsiung. In the study, the patients were divided into three age groups: 0–6 years (preschool), 7–12 years (primary school), and 13–15 years (junior high school). There were more hospitalizations due to asthma in Taipei than in Kaohsiung in each age group. The highest numbers of hospitalizations for asthma were in the groups aged from 0 to 6 years in both cities. There were more hospitalizations due to asthma of male patients than female patients in both cities. In terms of seasonal distribution, asthma hospitalizations in the two cities were concentrated in autumn (September, October, November) and winter (December, January, February), while the lowest incidence was seen in summer (June, July, August). 

### 3.2. Air Pollution Exposure

Table 2 shows the daily mean concentrations of ambient air pollutants during 2001–2010 in each city. The daily mean concentrations of ambient air pollutants during 2001–2010 in Kaohsiung were higher than those in Taipei, with the exception of NO_2_. In Taipei and Kaohsiung, respectively, the average concentrations were 27.53 and 46.84 μg/m^3^ for PM_2.5_, 47.13 and 77.49 μg/m^3^ for PM_10_, 26.98 and 29.27 ppb for O_3_, 3.61 and 7.82 ppb for SO_2_, and 23.35 and 22.27 ppb for NO_2_. These data indicated that air pollution in the heavily-industrial city of Kaohsiung was more severe than that in the business- and traffic-intensive city of Taipei. After season-stratified analysis, different concentrations of pollutants were observed in different seasons in the two cities: the PM_2.5_ concentration was higher in Kaohsiung in each season except for summer; the PM_10_ and SO_2_ concentrations were higher in Kaohsiung in all seasons; and the O_3_ and NO_2_ levels were higher in Taipei in spring and summer, and higher in Kaohsiung in autumn and winter. 

### 3.3. Air Pollution Change and Asthma Hospitalization

#### 3.3.1. Single-Pollutant Model of the Lagged Influence of Air Pollution on Asthma Hospitalization

Table 3 presents the results of analysis of the single-pollutant model in terms of the associations between air pollutants and the risk of childhood asthma hospitalization in both cities. No modification effect of season was observed after adjusting for daily mean temperature and relative humidity. SO_2_ was associated with childhood asthma hospitalization in Kaohsiung on lag day 1 (OR = 1.333, CI = 1.055–1.685). There were no significant associations between air pollution and asthma in Taipei.

According to age-stratified analysis (Table 3), in the 0–6 years age group, O_3_ was significantly positively associated with the timing of asthma admission in Taipei on lag day 3 (OR = 1.479, CI = 1.115–1.962), and SO_2_ was significantly positively associated with the timing of asthma admission in Kaohsiung on lag day 1 (OR = 1.595, CI = 1.177–2.163). In the 7–12 years age group, PM_10_ was significantly positively associated with the timing of asthma admission in Kaohsiung on lag day 1 (OR = 1.660, CI = 1.001–2.750). In the 13–15 years age group, O_3_ was significantly negatively associated with the timing of asthma admission in Kaohsiung on lag day 3 (OR = 0.098, CI = 0.015–0.646).

According to season-stratified analysis, in spring (Table 4), only the O_3_ level on the second day (OR = 1.646, CI = 1.008–2.688) and third day (OR =1.908, CI = 1.178–3.091) before asthma hospitalization exhibited a significant impact on asthma hospitalization in Taipei; there were no significant associations between the levels of PM_2.5_, PM_10_, SO_2_ or NO_2_ and asthma hospitalization in Taipei or Kaohsiung. In summer (Table 5), there were no significant associations between the levels of PM_2.5_, PM_10_, SO_2_, O_3_, or NO_2_ and asthma hospitalization in Taipei or Kaohsiung. In autumn (Table 6), PM_2.5_ was significantly associated with the timing of asthma admission on lag day 0 (OR = 0.765, CI = 0.607–0.963) and lag day 3 (OR = 0.749, CI = 0.595–0.9431) in Taipei. PM_10_ on lag day 0 (OR = 0.708, CI = 0.535–0.936) and lag day 3 (OR = 0.650, CI = 0.491–0.862) was significantly associated with childhood asthma hospitalization in Taipei, but not in Kaohsiung. NO_2_ was significantly associated with the timing of asthma admission on lag day 3 (OR = 2.395, CI = 1.044–5.491) in Kaohsiung. In winter (Table 7), only O_3_ was significantly associated with the timing of asthma admission on lag day 2 (OR = 0.433, CI = 0.217–0.862). PM_2.5_, PM_10_, SO_2_, and NO_2_ were not significantly associated with childhood asthma hospitalization in either city.

#### 3.3.2. O_3_, SO_2_, and NO_2_ Pollutants Adjusted for PM_2.5_, Temperature, and Relative Humidity

Because PM_2.5_ was highly-correlated with the other pollutants (Supplementary Table S1), autumn and winter were selected for the analysis of O_3_, SO_2_, and NO_2_ after controlling for PM_2.5,_ temperature, and relative humidity in the two cities. The results are shown in Table 8. After controlling for PM_2.5_, daily mean temperature, and relative humidity, the effect of NO_2_ in autumn was significantly associated with the timing of asthma admission on lag day 2 (OR = 1.942, CI = 1.155–3.265) and lag day 3 (OR = 2.054, CI = 1.242–3.397) in Taipei, and significantly associated with asthma hospitalization on lag day 3 (OR = 2.782, CI = 1.061–7.293) in Kaohsiung. In winter, O_3_ was significantly associated with asthma hospitalization on lag day 2 (OR = 0.437, CI = 0.219–0.872) in Taipei.

## 4. Discussion

This study compared the effect of exposure to air pollution on hospitalization due to childhood asthma in two cities in Taiwan with different urban patterns. This study comprehensively investigated the association between hospitalization due to childhood asthma and air pollution using a large-scale database. The results showed differing associations between asthma hospitalization in children and air pollution levels in two cities of Taiwan, Taipei, a business- and traffic-intensive city, and Kaohsiung, a large, heavily-industrial city, which are located in different geographical areas and have different climatic conditions. In this study, children aged 0–6 years had a higher rate of hospitalization due to asthma than children in the 7–12 and 13–15 years age groups. Aged-stratified analysis showed that the association between air pollution and childhood asthma hospitalization differs. Air pollutants have many effects on the health of both adults and children, but children’s vulnerability is unique [13]. Children are more likely to be sensitive at a young age [14], because only 80 percent of the alveoli in the lungs are formed after birth, and the lungs continue to change and develop through adolescence; lungs of very young children are highly vulnerable to damage [15]. We also found that there were more childhood hospitalizations of male patients than female patients in Taipei and Kaohsiung, a result consistent with previous studies performed in New York, Texas, Toyama (Japan), and the Basque region of Spain [4,15,16,17].

The major mechanisms of individual air pollutants responsible for triggering asthma exacerbations are thought to be associated with oxidative injury to the airways, leading to inflammation, remodeling, and an increased risk of sensitization [18].

Season-stratified analysis showed that the association between air pollution and childhood asthma hospitalization has seasonality, the largest effects being observed in spring in Taipei and in autumn in Kaohsiung. The NO_2_ level was higher in Kaohsiung in autumn, and was found to be associated with asthma hospitalization on lag day 3 in Kaohsiung, a finding consistent with previous reports. According to a review of 22 studies [19], NO_2_ showed a significant association with asthma exacerbation in children. In Fukuoka City, from 2001 to 2007, in children under 12 years of age, a 10 μg/m^3^ increase in NO_2_ on lag days 2–3 was significantly associated with an increase in asthma hospitalization [20]. In Taiwan, from 2001 to 2002, in patients aged <18 years, asthma hospitalization was significantly associated with seasonal changes in the concentrations of NO_2_, O_3_, SO_2_, and PM_10_, the most strongly correlated air pollutant variable being PM_10_, followed by O_3_ and SO_2_ [21]; however, that study did not distinguish between different regions and age groups. In our study, PM_10_ was significantly positively associated with the timing of asthma admission in Kaohsiung in the 7–12 years age group, but according to season-stratified analysis, PM_2.5_ and PM_10_ were negatively associated with asthma hospitalization in autumn in Taipei. In Toyama, Japan, from February to April, 2005 to 2009, a statistically significant association between asthma hospitalization and a heavy dust event was observed in children aged 1–15 years [17]. In a similar study, it was found that from 2006 to 2010 in Kaohsiung, higher levels of PM_2.5_ and PM_10_ enhanced the risk of hospital admission for asthma only on cool days (i.e., days with a mean temperature below 25 °C), with no significant associations being found on warm days (i.e., days with a mean temperature above 25 °C) [22]. In Taipei, from 2006 to 2010, increased asthma hospitalization was significantly associated with the PM_2.5_ level [23], but that study did not distinguish between different age groups. Our results were inconsistent before and after controlling for PM_2.5_ in autumn and winter, and variations in seasonal and regional effect estimates may partially arise from the chemical composition of particulate matter (PM). PM is a complex mixture of solid and liquid particles suspended in air. The size, chemical composition, and other physical and biological properties of particles vary with location and time [24]. This heterogeneity in PM components may cause different health effects through various pathways [25,26], and it has been suggested that there is a degree of heterogeneity in the effect of particulate matter on mortality within the same country [20].

Different air pollutants were associated with asthma in Taipei and Kaohsiung in children aged 0–6 years. O_3_ showed a significant association with asthma exacerbation only in children aged 0–6 years in Taipei, and SO_2_ showed a significant association with asthma exacerbation only in children aged 0–6 years in Kaohsiung. The main sources of SO_2_ in the developed world are primary emissions during energy production or industrial processes [18]. The heterogeneous results between cities could be due to Kaohsiung’s heavy industry. In fact, the SO_2_ concentration was higher in Kaohsiung in all seasons. According to a systematic review study, SO_2_ was significantly associated with asthma exacerbation in children aged 0–18 years [19]. In our study, O_3_ was positively associated with asthma hospitalization in children aged 0–6 years in Taipei, but a negative association with asthma hospitalization in 13–15-year-olds was observed in Kaohsiung. According to season-stratified analysis, O_3_ was positively associated with asthma hospitalization on lag days 2–3 in spring, but a negative association with asthma hospitalization was observed in winter in Taipei. Regarding the effects of O_3_ on childhood asthma hospitalization, previous studies have reported inconsistent results. In New York City, the risk of asthma hospitalization in 5–17-year-old girls was found to be significantly associated with O_3_ in the warm season (May–September), but a negative association was observed in boys aged 5–9 years, and O_3_ was not found to be associated with childhood asthma hospitalization in Canada [27]. In Basque country (a region of Spain), O_3_ was negatively correlated with childhood asthma, but was not correlated with adult asthma [16]. According to a review of 87 studies [28], O_3_ was found to be significantly associated with an increased risk of asthma-related hospitalization in 71 studies. Because the level of O_3_ is affected by sunlight, temperature, and other air pollutants, the relationship between the O_3_ level and childhood asthma hospitalization requires further research. 

The strength of this study was that it provided a long-term analysis of the risk of childhood asthma hospitalization in relation to air pollution in two cities of differing urban patterns, and the study findings can be generalized to other cities of similar urban natures. However, there were some limitations of our study. An exposure measurement bias was present, as we used the air pollutant concentrations measured at the monitoring station closest to the hospital to which a patient was admitted as a proxy of personal exposure, and thus these data did not represent the actual exposure of children with asthma. A series of studies suggested that risk estimates based on fixed-site ambient air pollution measurements are smaller than those estimated using personal measures [29]. It is therefore recommended that the actual exposure concentration be measured using personal devices in the future.

## 5. Conclusions

Our study, which was of a case-crossover design and controlled individual characteristics, demonstrated that children aged 0–6 years had a higher rate of hospitalization due to asthma than children of other ages. The associations between air pollutant concentrations and asthma hospitalization in children differed between the traffic-intensive city of Taipei and the heavily-industrial city of Kaohsiung in Taiwan. High levels of air pollution were found to have greater effects on childhood asthma in Kaohsiung than in Taipei after adjusting for seasonal variation. The results of our study suggested that measures should be taken to prevent asthma hospitalization in children aged 0–6 years in areas with high levels of O_3_ and SO_2_. The most important factor was O_3_ in spring in Taipei. In children aged 0–6 years, asthma was associated with O_3_ in Taipei and SO_2_ in Kaohsiung, after controlling daily mean temperature and relative humidity.

## Figures and Tables

**Table 1:** Hospitalization characteristics.

Variables	Taipei		Kaohsiung		*p*-Value
	*n* = 2900	%	*n* = 1337	%	
Age (years)					
0–6	2128	73%	897	67%	<0.001
7–12	689	24%	378	28%	
13–15	83	3%	62	5%	
Gender					
Male	2025	70%	886	66%	<0.001
Female	875	30%	451	34%	
Season					
Spring	798	27%	326	24%	0.217
Summer	452	16%	244	18%	
Autumn	845	29%	381	28%	
Winter	805	28%	386	30%	
Year					
2001	437	15%	258	19%	<0.001
2002	316	11%	140	11%	
2003	191	7%	127	9%	
2004	214	7%	144	11%	
2005	402	14%	126	9%	
2006	217	7%	102	8%	
2007	353	12%	96	7%	
2008	249	9%	112	8%	
2009	248	9%	101	8%	
2010	273	9%	131	10%	

**Table 2:** Summary statistics for air pollutants in Taipei and Kaohsiung, Taiwan, 2001–2010.

Pollutants	Taipei				Kaohsiung			
	Mean	SD	Median	IQR	Mean	SD	Median	IQR
PM_2.5_ (μg/m^3^)								
All	27.53	15.21	24.71	18.83	46.84	24.40	44.79	36.58
Spring	31.83	15.84	29.94	20.31	45.77	20.38	43.63	28.64
Summer	24.21	11.09	22.96	14.67	23.85	11.41	21.08	13.43
Autumn	25.08	14.86	21.71	17.00	51.93	20.86	50.88	27.00
Winter	29.73	17.34	26.83	23.29	65.62	22.18	63.63	28.46
PM_10_ (μg/m^3^)								
All	47.13	26.28	43.08	31.88	77.49	39.57	73.46	59.58
Spring	55.19	30.43	51.83	35.73	77.72	37.36	74.38	48.67
Summer	40.02	17.32	39.33	22.52	40.82	16.59	37.70	20.33
Autumn	42.18	22.82	38.38	27.92	85.83	34.72	84.11	47.79
Winter	50.88	29.25	45.96	41.27	106.11	34.04	104.75	44.92
O_3_ (ppb)								
All	26.98	13.19	24.81	16.90	29.27	13.46	27.83	19.05
Spring	32.38	14.62	30.10	18.66	31.90	14.10	30.85	20.82
Summer	23.34	11.41	21.91	15.08	22.85	11.14	20.47	14.33
Autumn	27.56	12.75	25.78	16.76	36.22	13.38	35.81	18.08
Winter	24.62	11.86	22.03	14.96	26.14	10.80	25.76	14.09
SO_2_ (ppb)								
All	3.61	2.25	3.24	2.70	7.82	5.23	6.70	6.13
Spring	3.77	2.30	3.43	2.89	7.90	4.76	7.04	5.88
Summer	3.72	2.00	3.46	2.50	5.83	4.46	4.88	4.78
Autumn	3.09	2.16	2.72	2.25	7.79	4.72	6.83	5.42
Winter	3.84	2.43	3.38	2.95	9.79	6.07	8.63	7.55
NO_2_ (ppb)								
All	23.35	13.07	23.94	16.69	22.27	11.25	20.71	16.16
Spring	26.72	14.31	27.07	17.29	21.86	9.67	20.71	13.45
Summer	20.63	10.64	21.51	15.37	13.20	6.13	12.67	8.12
Autumn	20.70	12.03	21.62	16.38	22.72	9.98	21.93	13.66
Winter	25.26	13.82	25.91	16.35	31.45	10.46	31.14	13.37

SD = standard deviation.

**Table 3:** Association between air pollution and childhood asthma.

Pollutants		No Modification Effect of Season					
		Taipei			Kaohsiung		
	Lag Day	OR	95% CI	*p*-Value	OR	95% CI	*p*-Value
All Subjects							
PM_2.5_	0	0.939	0.830–1.062	0.316	1.015	0.795–1.295	0.901
	1	0.941	0.841–1.052	0.289	1.099	0.856–1.410	0.457
	2	0.993	0.890–1.109	0.912	1.100	0.863–1.404	0.438
	3	0.954	0.848–1.075	0.445	1.127	0.880–1.444	0.340
PM_10_	0	0.935	0.826–1.060	0.298	1.100	0.915–1.322	0.309
	1	0.994	0.907–1.089	0.904	1.088	0.874–1.353	0.447
	2	1.027	0.928–1.136	0.603	1.037	0.826–1.300	0.752
	3	0.933	0.826–1.055	0.271	1.156	0.956–1.399	0.133
O_3_	0	0.858	0.666–1.105	0.236	1.008	0.817–1.243	0.936
	1	1.144	0.885–1.479	0.301	1.095	0.888–1.349	0.393
	2	1.047	0.807–1.359	0.725	1.068	0.862–1.322	0.546
	3	1.241	0.967–1.592	0.089	0.928	0.746–1.154	0.503
SO_2_	0	1.005	0.878–1.152	0.931	1.103	0.878–1.385	0.396
	1	1.010	0.876–1.164	0.884	1.333 *	1.055–1.685	0.015
	2	0.935	0.808–1.081	0.368	1.164	0.929–1.459	0.184
	3	0.962	0.833–1.111	0.600	1.059	0.840–1.335	0.625
NO_2_	0	1.148	0.938–1.405	0.178	1.026	0.737–1.426	0.877
	1	1.154	0.944–1.411	0.160	1.214	0.866–1.702	0.258
	2	1.190	0.966–1.467	0.101	1.179	0.832–1.670	0.353
	3	1.093	0.889–1.345	0.395	1.324	0.925–1.893	0.123
Group 1: 0–6 years							
PM_2.5_	0	0.939	0.816–1.081	0.383	0.992	0.748–1.317	0.960
	1	0.932	0.817–1.062	0.292	1.043	0.775–1.403	0.778
	2	0.981	0.867–1.109	0.762	1.237	0.927–1.651	0.147
	3	0.920	0.804–1.053	0.228	1.091	0.810–1.469	0.563
PM_10_	0	0.940	0.811–1.088	0.410	1.095	0.895–1.340	0.374
	1	0.997	0.894–1.111	0.960	1.012	0.795–1.288	0.920
	2	1.014	0.908–1.132	0.796	1.078	0.792–1.467	0.632
	3	0.908	0.790–1.043	0.173	1.117	0.882–1.414	0.357
O_3_	0	0.919	0.688–1.226	0.565	0.951	0.739–1.225	0.701
	1	1.031	0.770–1.382	0.834	1.052	0.825–1.343	0.677
	2	1.071	0.796–1.440	0.647	1.032	0.804–1.325	0.799
	3	1.479 **	1.115–1.962	0.006	1.037	0.801–1.341	0.781
SO_2_	0	1.054	0.907–1.225	0.488	1.059	0.810–1.385	0.673
	1	1.015	0.866–1.190	0.846	1.595 **	1.177–2.163	0.002
	2	0.888	0.752–1.049	0.165	1.292	0.969–1.723	0.080
	3	0.937	0.796–1.103	0.438	1.043	0.767–1.416	0.787
NO_2_	0	1.111	0.881–1.400	0.371	0.887	0.593–1.325	0.559
	1	1.135	0.904–1.423	0.272	1.145	0.761–1.723	0.513
	2	1.142	0.902–1.445	0.268	1.335	0.873–2.042	0.182
	3	1.040	0.822–1.316	0.738	1.329	0.856–2.063	0.203
Group 2: 7–12 years							
PM_2.5_	0	0.942	0.717–1.239	0.671	1.317	0.785–2.211	0.296
	1	0.951	0.755–1.198	0.673	1.456	0.886–2.392	0.137
	2	0.985	0.759–1.278	0.913	0.825	0.507–1.344	0.441
	3	1.006	0.766–1.320	0.963	1.353	0.837–2.187	0.216
PM_10_	0	0.932	0.723–1.201	0.588	1.369	0.831–2.254	0.216
	1	0.977	0.824–1.159	0.796	1.660 *	1.001–2.750	0.049
	2	1.018	0.774–1.338	0.895	1.005	0.713–1.417	0.975
	3	0.952	0.722–1.256	0.730	1.247	0.879–1.768	0.214
O_3_	0	0.747	0.429–1.300	0.302	1.126	0.741–1.711	0.576
	1	1.725	0.984–3.024	0.056	1.328	0.852–2.071	0.210
	2	0.957	0.540–1.697	0.882	1.367	0.868–2.150	0.176
	3	0.647	0.363–1.151	0.139	0.802	0.516–1.247	0.328
SO_2_	0	0.792	0.558–1.123	0.191	1.190	0.742–1.908	0.468
	1	0.911	0.643–1.292	0.604	0.938	0.631–1.394	0.753
	2	1.035	0.752–1.425	0.828	0.904	0.597–1.368	0.633
	3	1.002	0.726–1.382	0.988	1.052	0.712–1.554	0.797
NO_2_	0	1.308	0.838–2.043	0.236	1.713	0.908–3.231	0.096
	1	1.190	0.757–1.869	0.450	1.363	0.711–2.612	0.350
	2	1.394	0.868–2.239	0.168	0.864	0.441–1.692	0.671
	3	1.216	0.764–1.935	0.408	1.431	0.722–2.835	0.303
Group 3: 13–15 years							
PM_2.5_	0	0.930	0.317–2.726	0.894	0.154	0.019–1.235	0.078
	1	1.404	0.571–3.452	0.459	0.354	0.057–2.197	0.265
	2	2.118	0.881–5.089	0.093	0.622	0.131–2.955	0.550
	3	2.144	0.961–4.784	0.062	0.415	0.096–1.785	0.237
PM_10_	0	0.778	0.264–2.297	0.650	0.596	0.182–1.950	0.393
	1	1.616	0.620–4.210	0.325	0.426	0.059–3.043	0.395
	2	1.973	0.867–4.486	0.104	0.799	0.195–3.266	0.755
	3	2.012	0.876–4.620	0.099	0.727	0.135–3.894	0.710
O_3_	0	0.209	0.019–2.213	0.193	1.231	0.448–3.382	0.686
	1	0.631	0.067–5.917	0.687	0.769	0.244–2.417	0.653
	2	1.228	0.171–8.818	0.837	0.371	0.100–1.376	0.138
	3	0.680	0.097–4.738	0.697	0.098 *	0.015–0.646	0.015
SO_2_	0	0.632	0.164–2.435	0.505	1.547	0.427–5.601	0.505
	1	2.017	0.736–5.525	0.172	2.279	0.607–8.548	0.221
	2	2.466	0.779–7.802	0.124	1.882	0.387–9.133	0.432
	3	1.721	0.659–4.492	0.267	1.214	0.417–3.532	0.721
NO_2_	0	1.785	0.408–7.799	0.440	0.192	0.022–1.659	0.133
	1	3.286	0.433–24.94	0.249	2.823	0.405–19.67	0.294
	2	1.501	0.269–8.359	0.642	1.520	0.284–8.122	0.623
	3	2.853	0.648–12.55	0.165	0.755	0.126–4.519	0.758

Notes: * *p* < 0.05; ** *p* < 0.01.

**Table 4:** Association between air pollution and childhood asthma in spring.

Pollutants		Modification Effect of Season in Spring					
		Taipei			Kaohsiung		
	Lag Day	OR	95% CI	*p*-Value	OR	95% CI	*p*-Value
PM_2.5_	0	0.944	0.744–1.198	0.640	0.930	0.589–1.470	0.758
	1	0.859	0.708–1.043	0.125	1.681	0.999–2.827	0.050
	2	0.998	0.823–1.210	0.987	1.394	0.854–2.275	0.183
	3	0.955	0.770–1.183	0.675	1.599	0.940–2.719	0.083
PM_10_	0	0.859	0.706–1.047	0.133	1.084	0.867–1.356	0.475
	1	0.953	0.850–1.068	0.412	1.289	0.897–1.854	0.169
	2	0.989	0.863–1.134	0.882	1.231	0.853–1.776	0.265
	3	0.864	0.712–1.048	0.139	1.310	0.948–1.808	0.100
O_3_	0	0.724	0.457–1.145	0.167	1.011	0.685–1.494	0.953
	1	1.576	0.968–2.565	0.067	1.374	0.896–2.105	0.144
	2	1.646 *	1.008–2.688	0.046	1.337	0.851–2.099	0.206
	3	1.908 **	1.178–3.091	0.008	1.168	0.753–1.810	0.486
SO_2_	0	0.996	0.743–1.336	0.983	0.958	0.526–1.743	0.889
	1	0.921	0.683–1.242	0.590	1.455	0.855–2.474	0.166
	2	0.964	0.720–1.291	0.808	1.292	0.708–2.354	0.402
	3	1.064	0.807–1.403	0.655	1.313	0.767–2.247	0.320
NO_2_	0	1.283	0.885–1.859	0.187	0.731	0.390–1.371	0.329
	1	1.009	0.693–1.468	0.961	1.205	0.597–2.429	0.601
	2	1.060	0.722–1.558	0.763	1.244	0.622–2.487	0.536
	3	0.896	0.583–1.375	0.615	1.094	0.541–2.212	0.802

Notes: * *p* < 0.05, ** *p* < 0.01.

**Table 5:** Association between air pollution and childhood asthma in summer.

Pollutants		Modification Effect of Season in Summer					
		Taipei			Kaohsiung		
	Lag Day	OR	95% CI	*p*-Value	OR	95% CI	*p*-Value
PM_2.5_	0	0.952	0.616–1.470	0.824	0.460	0.155–1.362	0.161
	1	0.762	0.484–1.200	0.241	0.507	0.169–1.518	0.225
	2	0.838	0.541–1.297	0.428	0.632	0.233–1.712	0.367
	3	1.085	0.739–1.593	0.674	0.536	0.212–1.350	0.185
PM_10_	0	1.087	0.630–1.874	0.762	0.585	0.170–2.015	0.396
	1	1.212	0.709–2.073	0.481	0.324	0.090–1.160	0.083
	2	0.954	0.544–1.673	0.870	0.351	0.115–1.065	0.064
	3	1.146	0.695–1.889	0.592	0.375	0.123–1.138	0.083
O_3_	0	0.802	0.432–1.489	0.485	0.656	0.395–1.088	0.102
	1	0.874	0.471–1.622	0.671	0.677	0.401–1.144	0.145
	2	0.592	0.308–1.139	0.116	0.879	0.559–1.381	0.576
	3	0.741	0.420–1.306	0.300	0.726	0.447–1.177	0.194
SO_2_	0	0.827	0.536–1.276	0.392	1.154	0.751–1.774	0.511
	1	0.904	0.572–1.430	0.668	1.249	0.733–2.129	0.412
	2	1.067	0.717–1.588	0.748	1.164	0.729–1.858	0.524
	3	1.016	0.699–1.475	0.932	0.838	0.488–1.439	0.523
NO_2_	0	1.259	0.695–2.280	0.446	2.463	0.829–7.316	0.104
	1	0.961	0.536–1.724	0.895	1.677	0.594–4.732	0.327
	2	1.186	0.644–2.185	0.582	0.678	0.235–1.953	0.472
	3	1.055	0.599–1.857	0.851	1.268	0.432–3.721	0.665

**Table 6:** Association between air pollution and childhood asthma in autumn.

Pollutants		Modification Effect of Season in Autumn					
		Taipei			Kaohsiung		
	Lag Day	OR	95% CI	*p*-Value	OR	95% CI	*p*-Value
PM_2.5_	0	0.765 *	0.607–0.963	0.022	0.999	0.622–1.607	0.999
	1	0.966	0.787–1.186	0.745	0.969	0.592–1.586	0.901
	2	0.896	0.725–1.107	0.310	0.999	0.625–1.599	0.999
	3	0.749 *	0.595–0.943	0.013	1.117	0.704–1.771	0.637
PM_10_	0	0.708 *	0.535–0.936	0.015	1.000	0.596–1.676	0.999
	1	0.896	0.689–1.164	0.412	0.806	0.452–1.436	0.464
	2	0.805	0.611–1.060	0.123	0.999	0.578–1.728	0.999
	3	0.650 **	0.491–0.862	0.002	1.184	0.698–2.008	0.528
O_3_	0	1.166	0.752–1.809	0.490	1.256	0.835–1.889	0.272
	1	1.185	0.766–1.835	0.444	1.076	0.746–1.553	0.691
	2	1.288	0.834–1.988	0.252	1.000	0.675–1.481	0.999
	3	1.192	0.767–1.853	0.433	1.243	0.802–1.928	0.329
SO_2_	0	0.880	0.691–1.120	0.300	1.000	0.663–1.507	0.999
	1	1.025	0.796–1.318	0.847	1.261	0.839–1.896	0.263
	2	0.750	0.560–1.003	0.052	1.361	0.892–2.078	0.152
	3	0.877	0.663–1.161	0.362	1.274	0.803–2.022	0.303
NO_2_	0	0.888	0.600–1.314	0.552	0.999	0.493–2.027	0.999
	1	1.326	0.904–1.946	0.148	0.975	0.454–2.096	0.949
	2	1.372	0.904–2.081	0.136	1.000	0.455–2.196	0.999
	3	1.160	0.786–1.711	0.452	2.395 *	1.044–5.491	0.039

Notes: * *p* < 0.05, ** *p* < 0.01.

**Table 7:** Association between air pollution and childhood asthma in winter.

Pollutants		Modification Effect of Season in Winter					
		Taipei			Kaohsiung		
	Lag Day	OR	95% CI	*p*-Value	OR	95% CI	*p*-Value
PM_2.5_	0	1.101	0.876–1.383	0.406	1.361	0.901–2.056	0.142
	1	0.992	0.791–1.245	0.949	1.144	0.761–1.720	0.515
	2	1.060	0.858–1.309	0.587	1.230	0.823–1.837	0.311
	3	1.109	0.885–1.388	0.367	1.328	0.875–2.017	0.182
PM_10_	0	1.204	0.950–1.527	0.123	1.282	0.824–1.996	0.270
	1	1.104	0.881–1.383	0.387	1.175	0.767–1.798	0.457
	2	1.202	0.976–1.480	0.082	1.035	0.667–1.604	0.877
	3	1.210	0.962–1.522	0.102	1.181	0.754–1.850	0.465
O_3_	0	0.532	0.274–1.034	0.062	1.134	0.696–1.847	0.612
	1	0.589	0.302–1.150	0.121	1.620	0.983–2.668	0.058
	2	0.433*	0.217–0.862	0.017	1.309	0.797–2.148	0.286
	3	0.929	0.496–1.742	0.820	0.986	0.595–1.633	0.957
SO_2_	0	1.285	0.997–1.654	0.051	1.121	0.704–1.786	0.628
	1	1.119	0.870–1.439	0.377	1.427	0.913–2.231	0.117
	2	1.029	0.790–1.340	0.828	0.955	0.637–1.432	0.826
	3	1.022	0.776–1.346	0.873	0.915	0.608–1.379	0.674
NO_2_	0	1.346	0.891–2.032	0.157	1.323	0.759–2.305	0.322
	1	1.065	0.715–1.586	0.753	1.559	0.894–2.718	0.116
	2	1.011	0.673–1.518	0.955	1.458	0.819–2.594	0.199
	3	1.060	0.719–1.563	0.765	1.540	0.840–2.823	0.162

Note: * *p* < 0.05.

**Table 8:** Association between air pollution and childhood asthma in autumn and winter, adjusted for PM_2.5._

Pollutants	Lag Day	Taipei			Kaohsiung		
		OR	95% CI	*p*-Value	OR	95% CI	*p*-Value
**Autumn**							
O_3_	0	1.562	0.958–2.547	0.073	1.689	0.891–3.202	0.107
	1	1.252	0.784–1.998	0.345	1.183	0.661–2.116	0.570
	2	1.553	0.955–2.526	0.075	1.000	0.547–1.826	0.999
	3	1.607	0.989–2.611	0.055	1.323	0.696–2.518	0.392
SO_2_	0	0.976	0.759–1.254	0.851	1.051	0.874–1.265	0.590
	1	1.059	0.794–1.411	0.693	1.108	0.926–1.327	0.261
	2	0.746	0.527–1.055	0.097	1.148	0.951–1.386	0.148
	3	1.079	0.777–1.498	0.647	1.107	0.902–1.359	0.327
NO_2_	0	1.166	0.738–1.842	0.509	0.854	0.388–1.880	0.696
	1	1.525	0.976–2.381	0.063	0.990	0.428–2.285	0.981
	2	1.942 *	1.155–3.265	0.012	1.000	0.419–2.386	0.999
	3	2.054 **	1.242–3.397	0.004	2.782 *	1.061–7.293	0.037
**Winter**							
O_3_	0	0.537	0.276–1.044	0.067	0.919	0.617–1.707	0.919
	1	0.576	0.292–1.137	0.112	0.074	0.953–2.804	0.074
	2	0.437 *	0.219–0.872	0.018	0.433	0.730–2.080	0.433
	3	0.946	0.504–1.777	0.864	0.571	0.497–1.470	0.571
SO_2_	0	1.368	0.980–1.909	0.065	1.020	0.628–1.656	0.933
	1	1.216	0.878–1.683	0.238	1.408	0.883–2.245	0.150
	2	0.965	0.677–1.375	0.846	0.878	0.569–1.352	0.555
	3	0.892	0.615–1.294	0.547	0.841	0.549–1.288	0.426
NO_2_	0	1.323	0.823–2.124	0.246	1.053	0.529–2.098	0.881
	1	1.104	0.688–1.773	0.679	1.717	0.844–3.495	0.135
	2	0.938	0.583–1.508	0.792	1.367	0.661–2.829	0.398
	3	0.973	0.629–1.503	0.902	1.332	0.614–2.889	0.467

Notes: * *p* < 0.05, ** *p* < 0.01. Adjusted for PM_2.5_, temperature and relative humidity.

## Supplementary Materials

The following is available online at http://www.mdpi.com/1660-4601/15/4/647/s1. Table S1: Pearson correlation matrix of air pollutants.
