# Supplementary material for: Application of a Time-Stratified Case-Crossover Design to Explore the Effects of Air Pollution and Season on Childhood Asthma Hospitalization in Cities of Differing Urban Patterns: Big Data Analytics of Government Open Data
Source: Int J Environ Res Public Health. 2018 Mar 31;15(4):647. doi: 10.3390/ijerph15040647 (PMC5923689; doi:10.3390/ijerph15040647)
Supplement: Supplementary file 1 [file ijerph-15-00647-s001.pdf]

Table S1 Pearson correlation matrix of air pollutants and temperature in Taipei and Kaohsiung

**Taipei**	**$PM_{2.5}$**	**PM_{10}**	**SO_2**	**NO_2**	**O_3**	**Temperature**
$PM_{2.5}$	1					
PM_{10}	0.858**	1				
SO_2	0.545**	0.548**	1			
NO_2	0.492**	0.460**	0.461**	1		
O_3	0.367**	0.356**	0.072**	-0.039**	1	
Temperature	-0.105**	-0.163**	0.001	-0.245**	-0.064**	1
**Kaohsiung**	**$PM_{2.5}$**	**PM_{10}**	**SO_2**	**NO_2**	**O_3**	**Temperature**
$PM_{2.5}$	1					
PM_{10}	0.855**	1				
SO_2	0.443**	0.525**	1			
NO_2	0.768**	0.767**	0.705**	1		
O_3	0.372**	0.368**	-0.020	0.052**	1	
Temperature	-0.518**	-0.557**	-0.488**	-0.748**	0.136**	1

Note:**p-value ＜0.01
